# Efficacy and safety analysis of autologous hematopoietic stem cell transplantation for extraocular retinoblastoma

**DOI:** 10.3389/fped.2026.1810303

**Published:** 2026-04-29

**Authors:** Hongyan Liu, Suoqin Tang, Qi Liu, Yingli Zhang, Yukai Liu, Jianming Fang, Han Gong, Chiyu Wang, Zhouyang Liu, Yuan Sun

**Affiliations:** Department of Hematology and Oncology, Beijing Jingdu Children’s Hospital, Beijing, China

**Keywords:** autologous hematopoietic stem cell transplantation, central nervous system, metastatic retinoblastoma, safety, therapeutic effect

## Abstract

This study aimed to assess the efficacy and safety of autologous hematopoietic stem cell transplantation (Auto-HSCT) in pediatric patients with extraocular retinoblastoma. We performed a retrospective analysis of clinical data from 12 children diagnosed with extraocular retinoblastoma at our institution between September 2018 and February 2023. A conditioning regimen consisting of Etoposide, Thiotepa, and Carboplatin was administered to all patients, with individualized adjustments based on patient-specific characteristics. Notably, one patient with trilateral retinoblastoma who underwent salvage transplantation received a Melphalan/Busulfan-based conditioning regimen. All 12 patients successfully underwent stem cell mobilization, apheresis, and auto-HSCT, with no transplant-related mortality reported. The median follow-up duration after high-dose chemotherapy and auto-HSCT was 27 months (range, 2–59 months). During the follow-up period, 8 patients (66.7%) achieved sustained disease-free survival (DFS), whereas 4 patients (33.3%) died. Among the deceased, one case was attributed to disease progression in a patient with trilateral retinoblastoma. Stratified analysis demonstrated that the DFS rate was 50% (2/4) in patients with central nervous system (CNS) metastasis, in contrast to a significantly higher DFS rate of 85.7% (6/7) in those without CNS involvement. In conclusion, high-dose chemotherapy combined with auto-HSCT is an effective therapeutic strategy for extraocular retinoblastoma in children, characterized by reversible adverse events and favorable tolerability. Patients with retinoblastoma complicated by CNS metastasis exhibit a poor prognosis. For children with extraocular retinoblastoma without CNS involvement, auto-HSCT serves as a viable treatment option with potential curative efficacy.

## 1 Introduction

Retinoblastoma (RB) represents the most prevalent intraocular malignant tumor in children, with the majority of cases diagnosed before the age of two (1–3). In developing countries, RB patients present with advanced-stage disease due to delayed diagnosis and limited treatment options, resulting in persistently high morbidity and mortality rates. Traditional treatments, such as surgical resection and chemotherapy, commonly yield unsatisfactory outcomes, with poor prognoses in advanced-stage patients (4). Stem cell transplantation aims to leverage the regenerative capacity of stem cells to repair or replace damaged tissues or organs. This treatment modality holds significant potential for treating a range of diseases, demonstrating particular promise for hematologic disorders, specific cancers, and autoimmune disorders. Therefore, a significant number of researchers advocate stem cell transplantation following high-dose chemotherapy as a potential therapeutic strategy for patients with advanced or metastatic RB, potentially providing clinical benefits (5–7). Prior studies documented 3 of 5 patients with metastatic RB achieved post-transplant survival without evidence of central nervous system (CNS) involvement following sequential conventional therapy, high-dose chemotherapy, and autologous stem cell transplant (8). In our study, we collected clinical data from 12 children with extraocular retinoblastoma (EORB) treated at our hospital and assessed the efficacy and adverse effects of high-dose chemotherapy combined with autologous stem cell transplantation (HDCT-ASCT). Our findings provide a clinical basis for EORB treatment.

## 2 Patients and methods

### 2.1 Patients

A total of 12 EORB children were admitted to Department of Hematology and Oncology in Beijing Jingdu Children's Hospital from September 2018 to February 2023. Patients meeting the following criteria were included: (a) All children were diagnosed with EORB according to standard ophthalmologic and histological criteria. (b) These cases were classified according to the International Retinoblastoma Staging System (IRSS) (9). (c) Age < 18 years old. Children with irregular post-transfer treatment or incomplete treatment were excluded. The IRSS is specifically designed for patients with EORB who have developed distant metastasis. Stage 0 is characterized by completely resected intra-ocular tumors, as well as those with microscopic extra-ocular disease; Stage I by enucleation with complete histological resection; Stage II by enucleation but microscopic residual disease; Stage III involves overt regional extension (orbital or lymph node involvement); and Stage IV, defined by hematogenous metastases and those with CNS metastasis, representing a high-risk category with a very poor prognosis. All patients underwent ocular ultrasound, orbital CT, ocular MRI, fundus examination, and/or pathological examination, with RB confirmed. PET/CT, bone marrow aspiration, MRI, and cerebrospinal fluid cytology were used to assess tumor invasion, including involvement of the choroid, anterior chamber, sclera, iris and ciliary body, optic nerve, brain, and bone marrow. The patient cohort in this study comprised individuals diagnosed with Stage IV EORB. The study was approved by the Institutional Review Board of Jingdu Children's Hospital (Ethical Review Approval No. Jing Lun 2025-003-C). CNS metastasis was defined as the presence of either positive cerebrospinal fluid cytology or radiologically confirmed brain or leptomeningeal metastases on MRI.

### 2.2 Methods

The patients underwent HDCT-ASCT following more than 6 courses of systemic chemotherapy [VEC (Vincristine + Etoposide + Carboplatin) or IVE (Vincristine + Ifosfamide + Etoposide) regimen], surgery, and/or local therapy. The preparative regimen primarily consisted of Thiotepa, Carboplatin, and Etoposide. Other regimens could not be ruled out based on the child's specific clinical circumstances.

VEC (3 cases) or IVE (3 cases) chemotherapy, combined with recombinant human granulocyte colony-stimulating factor (rhG-CSF) and recombinant human granulocyte-macrophage colony-stimulating factor (GM-CSF), was used as the mobilization regimen. RhG-CSF and GM-CSF were administered subcutaneously daily until the day of autologous peripheral blood stem cell (PBSC) collection. The last dose was given 2 h prior to collection initiation. Administration continued until the white blood cell (WBC) count nadir was reached and subsequently rebounded to >2 × 10⁹/L. The total daily dose of rhG-CSF plus GM-CSF was 15 µg/kg/day with rhG-CSF administered subcutaneously in the morning and GM-CSF in the evening for 5 consecutive days. Once the WBC count exceeded 10 × 10^9^/L after day 5, PBSCs were collected using the COBE Spectra apheresis system. Collection continued until the cumulative CD34 + cell count exceeded 2 × 10^6^/kg. The collected PBSCs were cryopreserved at −196 °C (liquid nitrogen) at the Beijing Umbilical Cord Blood Bank. Prior to infusion, cells were thawed using a 37 °C water bath and administered intravenously. A comprehensive assessment of cardiac, pulmonary, hepatic, and renal function was performed before transplantation.

Preprocessing scheme (relative to Day 0 = stem cell infusion): Days −8 to −6: Carboplatin (500 mg/m²/day)-continuous intravenous infusion over 4 h with light protection; Days −5 to −3: Etoposide (VP-16) (250 mg/m²/day)-continuous intravenous infusion over 4 h, and Thiotepa (300 mg/m²/day)-intravenous infusion over 3 h. Days −2 to −1: Treatment rest; Day 0: Autologous PBSC infusion.

Prevention of post-transplant complications requires implementing key infection control measures, including environmental protection, basic nursing care, and topical medication. Additionally, rigorous oral care, anti-infection strategies, and immunologic support are essential to prevent mucosal injury, with timely protocol adjustments based on individual patient conditions. Throughout the ASCT phase, we performed regular monitoring for organ function and hepatic veno-occlusive disease (VOD).

Extramedullary toxicity was assessed using the regimen-related toxicity (RRT) grading criteria established by Beamall et al. (10). Eight organ systems—cardiac, renal, bladder, hepatic, pulmonary, central nervous system, gastrointestinal, and oral mucosa—were evaluated at days 0, 7, 14, and 28 post transplantation. Pulmonary RRT monitoring was extended to day 100. Myelosuppression severity was graded according to the Chinese Society of Clinical Oncology (CSCO) Expert Consensus on Diagnosis and Management of Antineoplastic Drug-Induced Myelosuppression (11).

### 2.3 Statistical analysis

Statistical analysis in this study was performed using SPSS 22.0, while data visualization was conducted with GraphPad Prism 9.0. Categorical data are presented as percentages (%). Survival analysis was performed using the Kaplan–Meier method to estimate disease-free survival (DFS), defined as the time from transplantation to disease recurrence, progression, or death, with statistical significance set at *p* < 0.05.

## 3 Result

**Figure 1:**
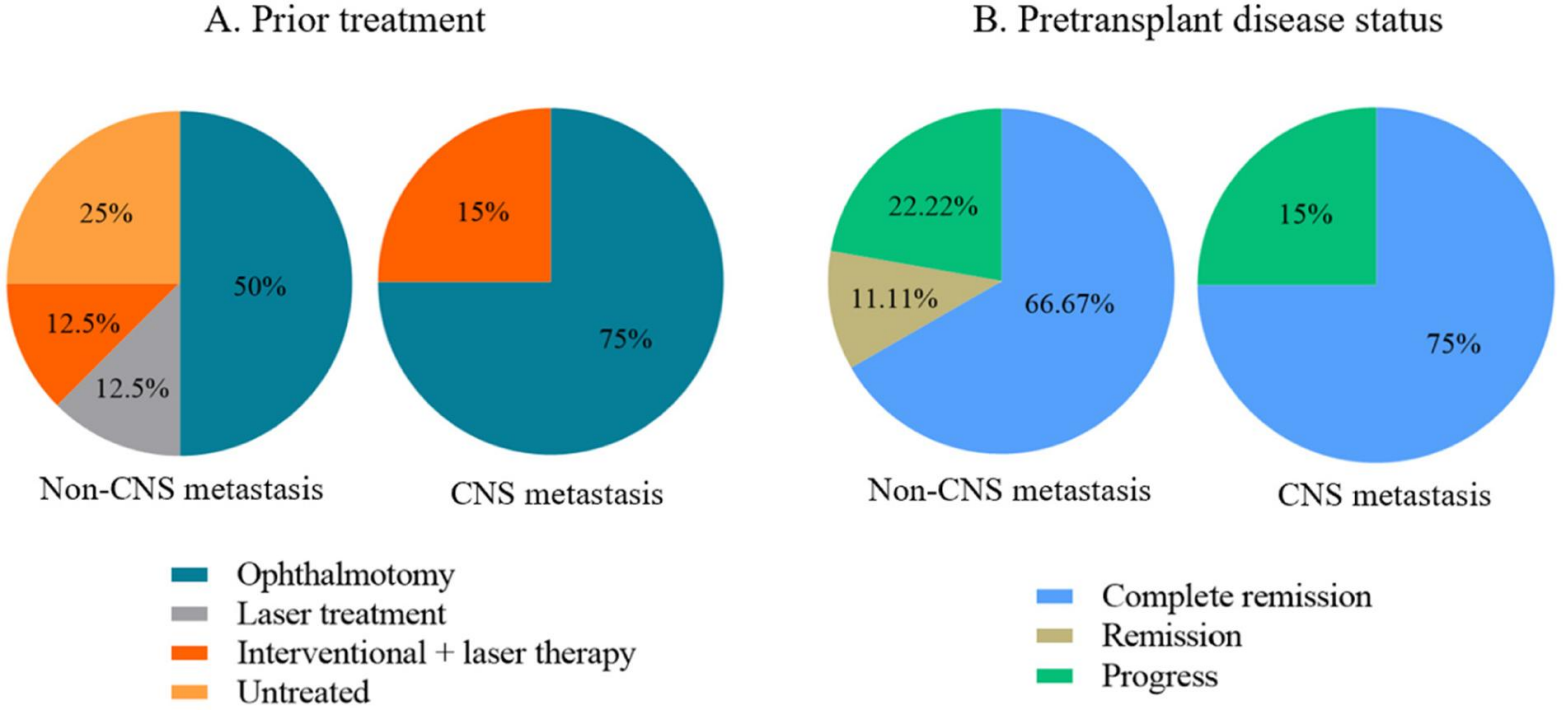
Treatment modalities **(A)** and disease status prior **(B)** to autologous stem cell transplantation.

The basic clinical data of patients were shown in Table 1. Among the 12 pediatric patients, 4 had CNS metastasis. One with trilateral retinoblastoma (RB) experienced disease progression following the first ASCT and subsequently underwent a second ASCT. As illustrated in Figure 1A, prior to ASCT, a total of 7 patients underwent enucleation, 2 received combined interventional and laser therapy, 2 received no prior treatment, and 1 received laser therapy alone. No patients received radiotherapy. All patients had undergone at least 6 cycles of intravenous chemotherapy prior to transplantation. The 7 patients underwent enucleation received the procedure after completion of induction chemotherapy but before high-dose chemotherapy combined with HDCT-ASCT. Before transplantation, 9 patients had achieved complete remission, while 3 had progressive disease. Including all trilateral RB patients achieved remission prior to the second transplant, as shown in Figure 1B. Histopathological examination of the enucleated specimens revealed high-risk pathological features in the majority of cases, including retrolaminar optic nerve invasion, choroidal invasion, involvement of the anterior chamber angle and ciliary body, and optic nerve thickening or intracranial enhancement. These features are consistent with the high-risk criteria defined in the International Retinoblastoma Staging System (IRSS) and the Children's Oncology Group ARET0321 trial, and are recognized as key predictors of extraocular dissemination and poor prognosis.

**Table 1:** Basic clinical data of patients with EORB.

Patient information	EORB children (*n*)
Age (month)	
Average value (SD)	20.3 (12.4)
Median (minimum, maximum)	20 (2, 49)
Gender	
Male	4 (33.3%)
Female	8 (66.7%)
Classification system	
D	1 (8.3%)
E	9 (75.0%)
Rests	2 (16.7%)
Pathogenic site	
Unilateral	9 (75.0%)
Ambilaterality	2 (16.7%)
Trilateral retinoblastoma	1 (8.3%)
Metastatic sites	
CNS metastasis	4 (33.3%)
Non-CNS metastasis	8 (66.7%)
Chemotherapy cycle	
Average (SD)	9.8 (2.7)
Median (minimum, maximum)	9 (7, 15)

As shown in Table 2, the mean number of reinfused CD34 + cells was 6.77 × 10^6^/kg, and the mean number of reinfused MNC cells was 6.17 × 10^8^/kg. The mean time to neutrophil engraftment was 9 days (range: 8–11 days), and the mean time to platelet engraftment was 11 days (range: 8–30 days).

**Table 2:** Stem cell transplantation.

SCT	Mean (Range)
Number of cells transfused (/kg)	
MNC	6.17 × 10^8^ (4.60 × 10^8^, 8.15 × 10^8^)
CD34+	6.77 × 10^6^ (1.78 × 10^6^,13.79 × 10^6^)
Implantation time (days)	
Neutrophil	9 (8, 11)
Platelet	11 (8, 30)

**Figure 2:**
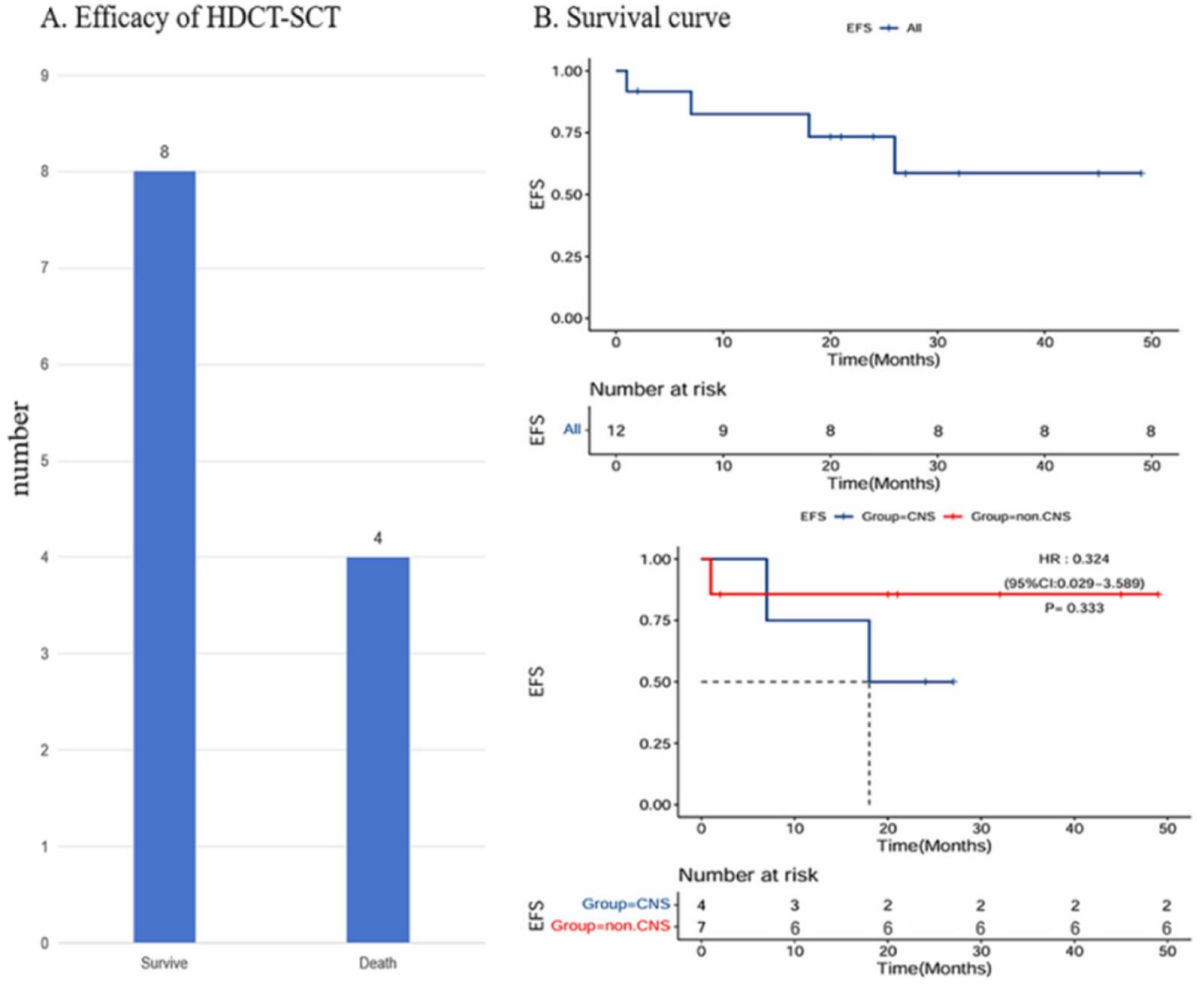
Efficacy assessment and prognostic analysis of autologous stem cell transplantation. [**(A)** presents a 2–59 months follow-up analysis of patients undergoing HDCT-ASCT. **(B)** compares the survival rates between pediatric patients with CNS metastasis and those without metastasis after receiving HDCT-ASCT].

The median follow-up duration after HDCT-ASCT was 27 months (range: 2–59 months). At the last follow-up date, 8 patients (66.7%) remained alive and disease-free. During the follow-up period after HDCT-ASCT, a total of 4 patient deaths were observed, including 2 cases of CNS metastasis-related deaths where patients succumbed to increased intracranial pressure and multi-organ failure due to extensive brain metastases, manifesting as progressive consciousness impairment and seizures (Figure 2A). 1 case of COVID-19-associated death with acute respiratory distress syndrome and thrombotic microangiopathy (TMA), characterized by severe pulmonary inflammation, thrombocytopenia, hemolytic anemia, and renal impairment leading to multi-organ failure; and 1 case of trilateral RB progression-related death where the patient with bilateral retinal tumors and pineal region brain tumor died from brainstem compression and hydrocephalus despite HDCT-ASCT, suggesting a potential clearance of CNS tumor cells. While this conversion to negative CSF status is encouraging, its clinical significance requires validation in larger cohorts with longer follow-up. The survival rate among patients with central metastasis was 50% (2/4), whereas those without central metastasis exhibited a significantly higher survival rate of 85.7% (6/7) after excluding trilateral RB cases. Notably, all 4 patients with initially positive cerebrospinal fluid (CSF) assessments achieved negative CSF status post-HDCT-ASCT, indicating effective clearance of CNS tumor cells by the treatment regimen. Although the intergroup survival difference did not reach statistical significance (*p* > 0.05), Figure 2B demonstrates a clinically relevant trend: children without central metastasis who underwent HDCT-ASCT had superior survival outcomes compared to those with central involvement.

The adverse reactions observed during the treatment course were predominantly myelosuppression and gastrointestinal toxicity, with mucosal injuries mostly being mild. Among the 12 cases, 7 patients did not develop any form of infection (including one case of second trilateral RB autologous stem cell transplantation), 5 cases developed pulmonary infections, and 1 case exhibited infection at the infusion port. TMA occurred in only 1 of the 12 cases, as detailed in Table 3. All pediatric patients (12/12) developed febrile symptoms at (7 ± 2) days post-transplantation, with body temperatures ranging from 38.5 ℃ to 39.5 ℃. Notably, these episodes were not accompanied by chills, and blood culture results were negative. Following treatment with carbapenems and vancomycin, the infections were effectively controlled, with no significant hemorrhagic tendencies observed. One patient experienced septic shock with hypotension requiring vasopressors and febrile neutropenia. All blood cultures remained negative, a common finding in heavily immunocompromised transplant recipients. The patient recovered hemodynamically within 48–72 h after empirical antibacterial therapy, consistent with clinically presumed sepsis (12, 13).

**Table 3:** Treatment-related adverse events and toxicities by patient.

Patient's code	Myelosuppression	Mucosal injury	Gastrointestinal toxicity	Urotoxicity	Infection	TMA
P01	IV	II	III	0	Pulmonary infection	N
P02	IV	III	III	II	N	N
P03	IV	II	III	III	Pulmonary infection	Y
P04	IV	II	III	0	Pulmonary infection	N
P05-1	IV	III	III	0	Pulmonary infection	N
P05-2	IV	0	II	0	N	N
P06	IV	III	III	0	Infusate-related bloodstream infection	N
P07	IV	II	III	0	N	N
P08	IV	II	III	0	N	N
P09	IV	II	III	0	N	N
P10	IV	III	III	0	Pulmonary infection	N
P11	IV	III	III	0	N	N
P12	IV	II	III	0	N	N

## 4 Discussion

RB metastasis is a significant cause of mortality in RB patients. In this context, HDCT-SCT has emerged as a promising therapeutic strategy (14–17). A statistical analysis by Audrey et al. (18) revealed that among 160 patients treated with high-dose chemotherapy (HDCT) combined with stem cell transplantation (SCT), 108 patients (67.5%) achieved event-free survival. Another study by Imad et al. (4), among 101 patients receiving HDCT-SCT, 44 out of 77 patients (57.1%) with relapse or metastasis survived. Notably, the recurrence rate was 73.1% in patients with central nervous system (CNS) metastasis, which decreased to 47.1% following thiotepa treatment. Supporting these findings, Nan et al. (19) reported a cohort of 16 metastatic RB patients, including 8 children (50%) with CNS metastasis [2 (12.5%) with parenchymal brain metastasis and 6 (37.5%) with optic chiasm metastasis] and 8 (50%) with metastasis to other tissues (bone, parotid gland, lymph nodes, and bone marrow). All patients remained event-free during the follow-up period. These findings suggest that high-dose chemotherapy followed by stem cell transplantation may be effective and well-tolerated in RB patients. Long-term tumor control can be achieved in patients without CNS metastasis. For patients with CNS metastasis, Thiotepa-based chemotherapy may contribute to improved tumor control (4, 19). Furthermore, tandem high-dose chemotherapy followed by autologous stem cell transplantation has been reported as a feasible salvage approach for patients with ultra-high-risk metastatic or trilateral retinoblastoma, particularly those with CNS metastasis. Toret et al. described a successful case of an infant with trilateral retinoblastoma and CNS metastasis who achieved disease control after conventional chemotherapy followed by tandem HDCT and tandem autologous HSCT, supporting the potential role of repeated intensive consolidation therapy in this poor-prognosis population (20). In addition, Sait et al. demonstrated that HDCT with autologous stem cell rescue, combined with craniospinal irradiation or intraventricular radioimmunotherapy, could improve outcomes in patients with CNS retinoblastoma, further supporting the value of intensive consolidation strategies in this poor-prognosis population (18).

Dunkel et al. (21) reported an approach for treating IVa/IVb EORB patients involving 4 cycles of induction chemotherapy, followed by high-dose carboplatin with autologous hematopoietic stem cell support for partial responders and radiotherapy for those with residual tumor, which yielded 1-year event-free survival rates of 82.6% and 28.3% for Stage IVa and IVb patients, respectively. In this study, after excluding cases with trilateral RB involving intraventricular involvement, patients with CNS metastasis exhibited a survival rate of 50%, while those without CNS metastasis achieved a significantly higher survival rate of 85.7%, demonstrating a substantial improvement compared to conventional therapy. The treatment strategy employed in this study-combining intrathecal chemotherapy with post-transplant maintenance chemotherapy—demonstrates significant advantages. Intrathecal chemotherapy directly penetrates the blood-brain barrier and blood-ocular barrier, achieving high therapeutic drug concentrations in the cerebrospinal fluid. This effectively targets and eradicates CNS metastatic foci that are often inaccessible to conventional therapies. Concurrently, maintenance chemotherapy provides sustained suppression of residual tumor cell proliferation, substantially reducing the risk of disease relapse. Furthermore, this combined approach circumvents the structural damage associated with surgery and radiation, leading to improved globe preservation rates and enhanced quality of life for pediatric patients.

The disparity in treatment efficacy is inherently associated with metastatic site. Studies have shown that patients with non-CNS metastasis exhibit more effectively treatment response compared to those with CNS involvement, potentially attributable to restricted blood-brain barrier permeability of chemotherapeutic agents (8). As an alkylating agent, Thiotepa effectively penetrates the blood-brain barrier, demonstrating therapeutic utility for RB patients with CNS metastasis. Similarly, chemotherapy regimens incorporating high-dose etoposide prove critical for stage IV metastatic RB (21, 22). In our cohort of 12 children with advanced metastatic disease, CNS metastasis was present in four cases, with three fatalities (including one trilateral retinoblastoma case) following HDCT-ASCT, consistent with existing literature (16). Our findings further indicate that HDCT-ASCT provides only transient benefit and poor overall prognosis for CNS metastasis patients. Conversely, HDCT-ASCT delivers reliable therapeutic outcomes for non-CNS metastatic pediatric patients, with manageable adverse effects and favorable safety profiles. While HDCT-SCT application for advanced relapsed/metastatic RB remains debated, our study corroborates evidence that patients with non-CNS metastasis derive substantial benefit from this treatment (5, 6, 23).

The tolerability of the HDCT-ASCT regimen is a critical consideration, with reported toxicity profiles generally falling within manageable ranges. Key studies, including one by Lee et al., have reported the absence of severe complications such as TMA in pediatric patients undergoing autologous HDCT-ASCT. Notably, these treatments have allowed for the avoidance of external beam radiotherapy in some cases, thereby preserving visual function—with some children maintaining normal vision in one or both eyes—and potentially reducing the risk of secondary malignancies in the long term (24). This favorable safety record is further corroborated by case reports, such as that from Hertzberg et al., which described successful HDCT-ASCT administration without significant adverse reactions, leading to sustained remission for over four years without the need for radiotherapy (25). This finding aligns with outcomes in our cohort of 12 extraocular RB patients, wherein 6 of 7 children (85.7%) with non-CNS metastasis survived to follow-up. Such evidence substantiates Guillermo et al.'s earlier assertion that intensive consolidation therapy mitigates metastasis risk—a recognized pathological determinant in unilateral retinoblastoma (26). Collectively, the evidence indicates that HDCT-SCT can facilitate sustained disease control during remission with an acceptable toxicity burden, particularly for patients without CNS metastases. However, the application of this intensive therapy for intraocular RB requires further clinical investigation to fully define its role and optimize patient selection.

In summary, the treatment of late-relapsed or metastatic RB remains a key focus of current research. This retrospective homogeneously treated large institutional-cohort study enrolled 12 children with metastatic RB and demonstrated that HDCT-SCT significantly improved treatment response rates with good tolerability. Further validation of its clinical value through large-scale collaborative studies is warranted.

## Data Availability

The raw data supporting the conclusions of this article will be made available by the authors, without undue reservation.
